# The Texas State Board of Medical Examiners

**Published:** 1901-07

**Authors:** 


### TEXAS MEDICAL JOURNAL.

AUSTIN, TEXAS.

A MONTHLY JOURNAL OF MEDICINE AND SURGERY.

EDITED AND PUBLISHED BY
F. E. DANIEL, M. D.

ASSOCIATE EDITOR:
WITTEN BOOTH RUSS, M. D.,
San Antonio, Texas.

Published Monthly at Austin, Texas. Subscription price $1.00 a year in advance.

Eastern Representative: John Guy Monihan, St. Paul Building, 220 Broadway, New York City.

Official organ of the State Association of Health Officers, the West Texas Medical Association, the Houston District Medical Association, the Austin District Medical Society, the Brazos Valley Medical Association, the Galveston County Medical Society, and several others.

### THE TEXAS STATE BOARD OF MEDICAL EXAMINERS.

Texas has her first State Board of Medical Examiners. She has also her first State Board of Homeopathic Medical Examiners and State Board of Eclectic Medical Examiners. The three boards make one, a kind of Trinity, of fish, flesh and fowl. An applicant for license pays his money and takes his choice. He can select the board to do the examining; but failing to pass he cannot go before either of the other boards.

The new medical practice law went into effect at noon July 8 (inst.), and on the following day the Governor announced the following appointments, nine to each board, selected from lists of eighteen names submitted by each of the three State associations:

BOARD OF MEDICAL EXAMINERS FOR THE STATE OF TEXAS.

J. W. Scott, M. D. . . . . Houston.
J. H. Evans, M. D. . . . . Palestine.
D. J. Jenkins, M. D. . . . . Daingerfield.
J. T. Wilson, M. D. . . . . Sherman.
M. M. Smith, M. D. . . . . Austin.
John C. Jones, M. D. . . . . Gonzales.
J. H. Reuss, M. D. . . . . Cuero.
Frank Paschal, M. D. . . . . San Antonio.
Sam R. Burroughs, M. D. . . . . Buffalo.

BOARD OF HOMEOPATHIC MEDICAL EXAMINERS FOR THE STATE OF TEXAS.

Geo. D. Streeter, M. D.....Waco.
Joseph R. Pollock, M. D.....Fort Worth.
William R. Owen, M. D.....San Antonio.
A. O. Buck, M. D.....Corsicana.
M. S. Metz, M. D.....McKinney.
Geo. E. Thornhill, M. D.....Paris.
William T. Smith, M. D.....Denison.
N. O. Brenizer, M. D.....Austin.
T. J. Crowe, M. D.....Dallas.

BOARD OF ECLECTIC MEDICAL EXAMINERS FOR THE STATE OF TEXAS.

G. W. Johnson, M. D.....San Antonio.
G. Helbing, M. D.....Bonham.
E. L. Fox, M. D.....Houston.
C. D. Hudson, M. D.....Speegleville.
J. N. White, M. D.....Queen City.
L. S. Downs, M. D.....Galveston.
W. J. Bell, M. D.....Gainesville.
N. V. Mitchell, M. D.....Dallas.
Charles Dowdell, M. D.....Ennis.

The appointees will meet at an early day and organize the respective boards. The time and place of first meeting will be duly announced through the State papers and through the several medical journals.

